# Dietary supplementation of garlic leaf extract enhances growth performance and intestinal health, immunity, and gut microbiota in broiler chickens

**DOI:** 10.1016/j.psj.2026.107651

**Published:** 2026-08-18

**Authors:** Sarana Rose Sommano, Piyachat Sunanta, Chiao-Hsu Ke, Yu-Lei Wang, Raktham Mektrirat, Aye Chan Moe, Achmad Muzakky Dityana, Chompunut Lumsangkul

**Affiliations:** a Bioactive Compound Laboratory, Faculty of Agriculture, Chiang Mai University, Chiang Mai, 50200, Thailand; b Multidisciplinary Research Institute, Chiang Mai University, Chiang Mai, 50200, Thailand; c Department of Veterinary Medicine, School of Veterinary Medicine, National Taiwan University, Taipei City 106, Taiwan; d Veterinary Academic Office, Faculty of Veterinary Medicine, Chiang Mai University, Chiang Mai, 50100, Thailand; e Chinese–Thai Collaborative Laboratory for Veterinary Acupuncture and Herbal Pharmacotherapy, Faculty of Veterinary Medicine, Chiang Mai University, Chiang Mai, 50100, Thailand; f Center of Excellence in Pharmaceutical Nanotechnology, Faculty of Pharmacy, Chiang Mai University, Chiang Mai, 50100, Thailand; g Monogastric Immunonutrition Laboratory, Department of Animal Science, National Chung Hsing University, Taichung, 402, Taiwan; h International Master Program of Agriculture, National Chung Hsing University, Taichung, 402, Taiwan; i International Doctoral Program in Agriculture, College of Agriculture and Natural Resources, National Chung Hsing University, Taichung, Taiwan; j Department of Animal Science, National Chung Hsing University, Taichung, 402, Taiwan; k The iEGG and Animal Biotechnology Research Center, National Chung Hsing University, Taichung, 402, Taiwan

**Keywords:** Garlic leaf extract, Broiler chicken, Immune response, Gut health, Gut microbiota

## Abstract

The poultry industry has increasingly sought natural feed additives as alternatives to synthetic growth promoters to enhance productivity and animal health in a sustainable manner. Garlic and its bioactive compounds have been widely recognised for their antimicrobial, antioxidant, and immunomodulatory properties, yet limited information is available regarding the efficacy of garlic leaf extract (**GLE**) in broiler diet. This study evaluated the effects of GLE as a natural dietary additive on growth performance, intestinal morphology, immune response, and gut microbiota composition in broiler chickens. A total of 160 one-day-old broilers were randomly assigned to 4 dietary treatments supplemented with 0, 125, 250, or 500 mg/kg of GLE for 35 days. Results revealed that dietary inclusion of 125 mg/kg GLE significantly increased final body weight and average daily gain compared with the control group. Supplementation with GLE also improved villus height in the jejunum and ileum suggesting enhanced nutrient absorption and intestinal integrity. The GLE treatment group exhibited significantly greater than that observed in the other experimental groups. In contrast, the concentrations of IgM and IgY did not differ significantly among treatments. Analysis of 16S rRNA gene sequences indicated that dietary GLE significantly altered the composition and structure of the intestinal microbiota, characterised by an increased relative abundance of *Firmicutes*, a reduction in *Bacteroidetes,* and enrichment of beneficial genera such as *Lactobacillus*. Principal coordinate and LEfSe analyses confirmed these compositional shifts. In conclusion, dietary inclusion of garlic leaf extract, particularly at 125 mg/kg, effectively enhanced growth performance, intestinal morphology, immunity, and gut microbial balance, supporting its potential as a natural feed additive in broiler production.

## Introduction

The routine and unregulated use of in-feed antibiotic growth promoters (**AGPs**) in the poultry industry has been linked to the emergence of antimicrobial resistance and harmful drug residues, posing a severe threat to global human, animal, and environmental health (Chen, et al., 2021; Kothari, et al., 2019). Consequently, the ban on sub-therapeutic antibiotics by regulatory bodies has intensified the search for effective, safe, and natural phytogenic feed alternatives that can maintain broiler productivity without compromising health (Demir, et al., 2005). Among various phytogenic candidates, garlic (Allium sativum) is globally recognised for its profound medicinal and therapeutic properties (Somamno et al., 2016). The beneficial effects of garlic are largely attributed to its rich composition of bioactive molecules, primarily organosulfur compounds such as allicin, alliin, diallyl disulfide, and diallyl trisulfide, alongside polyphenols, saponins, and fructo-oligosaccharides (Kothari et al., 2019; Sunanta et al., 2023b). Extensive research has validated the efficacy of garlic bulb derivatives such as powders and extracts, as dietary supplements in broilers, demonstrating significant improvements in body weight gain, feed conversion ratios, immune responses, and gut histomorphology (Ari et al., 2012; Elagib et al., 2013; ELTAZI et al., 2014; Mufungwe et al., 2024). Furthermore, garlic supplementation effectively modulates the intestinal microbiota by suppressing pathogenic bacteria and promoting beneficial flora, thereby enhancing overall gut health (Chen et al., 2021; Kothari et al., 2019).

Despite the extensive utilisation of garlic bulbs, the commercial garlic peeling and processing industries generate massive amounts of agricultural by-products annually, primarily consisting of leaves, stems, and skins, which can account for more than 10% to 30% of the total dried weight (Sunanta, et al., 2023a; Sunanta, et al., 2024a). Historically, these by-products are frequently discarded, burned, or improperly managed, leading to severe environmental pollution, emission of air pollutants, and increased operational management costs (Sunanta, et al., 2023a). Aligning with the Sustainable Development Goals (SDGs) and the bio-circular green economy model, recovering these agricultural biomasses represents a highly valuable and untapped strategy for extracting functional bioactive ingredients (Khamsaw, et al., 2022). Utilising these by-products not only reduces waste but also establishes a sustainable supply chain for the food and agricultural sectors (Khamsaw et al., 2022; Sunanta et al., 2024a).

While the phytochemical profile of garlic leaves indicates they possess potent bioactive properties, including high phenolic and flavonoid contents and significant antioxidant capacity, their specific application as a formulated dietary additive in poultry remains largely underexplored. The vast majority of existing literature focuses heavily on garlic bulbs or powders (Ari et al., 2012; Elagib et al., 2013; ELTAZI et al., 2014; Mufungwe et al., 2024), creating a distinct knowledge gap regarding the efficacy, safety, and optimal inclusion levels of garlic leaf extract (**GLE**) in broiler diets. Furthermore, a comprehensive understanding of the holistic biological mechanisms of GLE in broilers is still lacking, particularly concerning how varying doses of GLE influence intestinal barrier integrity, modulate immune- and antioxidant-related gene expression in vital organs, and reshape the cecal microbial community structure. Therefore, this study evaluates the effects of varying dietary inclusion levels of GLE on the growth performance, intestinal morphology, systemic immunity, and gut microbiota of broiler chickens. By addressing these gaps, this research aims to provide a sustainable, bio-circular solution for agricultural waste while establishing a highly effective natural alternative to synthetic AGPs in modern poultry production.

## Material and methods

### Garlic leaves extraction and chemical characterization

#### Plant material

During commercial processing, the garlic biomass generated by the garlic peeling industry in the Mae Tang district of Chiang Mai, Thailand, was monitored and evaluated (Sunanta, et al., 2024a). To prepare the samples, garlic leaves were separated from the biomass, cleaned with tap water, and dried overnight at 50°C until a constant weight was achieved. Before further usage, the samples were grinded to finely powder and stored in an airtight container.

#### Garlic leaf extract

The preparation of garlic leaf biomass was adapted from previously described methods (Sunanta et al., 2024a). The leaf powder sample was macerated for 24h in 85% (v/v) ethanol at room temperature at a ratio of 1:10 (w/v), using solvent extraction principles established for the recovery of garlic bioactives (Sunanta et al., 2023b). Following that, the extraction solutions were filtered using Whatman No. 1 filter paper (Whatman Ltd., England). The solid part was re-macerated 3 times. The supernatant was subsequently evaporated to dryness and stored at -20°C. Before analysing the chemical characteristics, the evaporated sample generally known as GLE was re-dissolved in 85% (v/v) ethanol at a concentration of 100 mg/mL, an approach similarly used for determining the phytochemical profiles of garlic extracts (Sunanta et al., 2020).

#### Chemical properties

##### Reducing sugar content

Total reducing sugars were quantified using the 3,5-dinitrosalicylic acid (**DNS**) assay adapted from Sunanta et al. (2023a), with standard glucose. In brief, 100 µL of the diluted GLE was reacted with 300 µL of DNS solution at 80°C for 30 minutes. Following incubation, absorbance was recorded at 575 nm via spectrophotometry, and the results were expressed as milligrams of reducing sugar per gram of crude extract.

##### Total phenolic content

Following the method of Sunanta et al. (2021), the total polyphenol content of GLE using gallic acid as a standard was determined. Primarily, 30 µL of the diluted GLE was combined with 150 µL of Folin-Ciocalteu reagent, followed by 120 µL of 7.5% w/v NaCO3 solution. The reaction was initiated in the darkness at room temperature for 60 min. A spectrophotometer was then used to determine the wavelength at 765 nm, and the total polyphenol content was expressed in milligrams of gallic acid equivalents (**GAE**) per gram crude extract.

##### Total flavonoid content

The content of total flavonoids was determined using catechin as a standard (Sunanta et al., 2020). The GLE solution (25 µL) was added to 125 µL of distilled water, followed by the addition of 7.5 µL of a 5% NaNO_2_ solution. After allowing the mixture to react at room temperature for 5 minutes, 15 µL of 10% AlCl3•6H2O solution was added. After 6 min, 1M of NaOH (50 µL) and distilled water (27.5 µL) were added. The absorbance at 510 nm was measured using a spectrophotometer. The total flavonoid content was expressed as mg Catechin Equivalents (CE) per gram crude extract.

##### DPPH (2,2-diphenyl-1-picrylhydrazyl) scavenging radical activity

Antioxidant capacity was determined using the DPPH assay as described by Sangta, et al. (2021). In brief, 25 µL of the extract was reacted with 250 µL of a 0.2 mM DPPH ethanolic solution (prepared by dissolving 0.005 g of DPPH in 100 mL of ethanol). Following 30 minutes of dark incubation at room temperature, the absorbance was recorded at 550 nm via spectrophotometry. The resulting antioxidant activity was expressed both as a percentage of DPPH decolorization and as milligrams of Trolox Equivalents (TE) per gram of crude extract.

##### ABTS [2,2′-azino-bis(3-ethylbenzothiazoline-6-sulfonic acid)] scavenging radical activity

The ABTS radical scavenging activity was evaluated following previously described protocols (Khamsaw et al., 2024). A 7 mM stock solution was prepared by dissolving 8 mg of ABTS in distilled water. Subsequently, potassium persulfate was added to the stock solution, and the mixture was allowed to stand in the dark for 12–16 h at 25°C to generate the ABTS radical cation. For the assay, 10 µL of the sample extract was mixed with 200 µL of the ABTS working solution and incubated for 30 minutes at room temperature. The absorbance was then measured at 734 nm using a spectrophotometer. The antioxidant capacity was quantified as the percentage of ABTS decolourisation and expressed as milligrams of Trolox Equivalents (TE) per gram of crude extract.

##### Quantitative determination of polyphenols

For this purpose, the GLE was prepared at 1 mg/mL in 85% ethanol and filtered through a 0.22 µm nylon filter prior to analysis. High-Performance Liquid Chromatography (HPLC) was conducted using a Chromaster 5000 system (HITACHI, Tokyo, Japan), adapting the methodology previously described by Sangta et al. (2021); Sunanta et al. (2024b). The system was equipped with a COSMOSIL C18 column (250 × 4.6 mm, 5 µm; Kyoto, Japan), with auto-sampler, and a Chromaster 5430 diode array detector. Chromatographic separation was achieved using a binary mobile phase consisting of 0.1% formic acid in distilled water (Mixture A; 0.1:99.9, v/v) and a blend of acetonitrile, formic acid, and distilled water (Mixture B; 94.9:0.1:5, v/v/v). Gradient elution was performed at a flow rate of 1 mL/min with an injection volume of 10 µL. The 20-minute gradient program began at 80% A, decreased to 30% A over 3 minutes, and was held for 1 minute. The composition was then increased to 55% A over 1 minute, rapidly reduced to 10% A, and subsequently raised to 60% A over 3 minutes, followed by a 3-minute hold. Finally, the mobile phase was returned to 80% A over 3 minutes and maintained until the end of the analysis. All samples were analysed in triplicate, with chromatograms recorded at 260 nm. Quantification was performed using calibration curves generated from a mixed standard solution of ten polyphenols (gallic acid, mangiferin, catechin, epicatechin, caffeic acid, rosmarinic acid, quercetin, lutein, vanillic acid, and p-coumaric acid) serially diluted at concentrations ranging from 0 to 100 ppm.

### Animal husbandry and feeding

The animal study protocol was conducted in strict accordance with the guidelines recommended and ethically approved by the Institutional Animal Care and Use Committee, National Chung Hsing University with the protocol number 114-042, and all efforts were made to minimize bird discomfort. The study was conducted at the Animal Experimental Farm, National Chung Hsing University, using a completely randomised block design. A total of 160 one-day-old straight-run (mixed-sex) Ross 308 broiler chicks were randomly allocated to 20 floor pens bedded with rice husks, with 8 birds per pen, providing 5 replicates per treatment. The experimental diets included: (1) a basal diet (**Control**), (2) basal diet supplemented with 125 g GLE/kg (**GLE125**), (3) basal diet supplemented with 250 g GLE/kg (**GLE250**), and (4) basal diet supplemented with 500 g GLE/kg (**GLE500**). The nutrient composition of the basal diets for the starter (between day 1 to 21) and finisher (between day 22 to 35) phases is presented in Table 1. Feed and water were provided ad libitum throughout the 35-day experimental period. Birds were reared at a stocking density of 7 birds/m² in a conventional open-sided house equipped with side curtains and ventilation fans. Environmental conditions were maintained at 20 ± 3°C, relative humidity of 65–75%, with a 12-hour photoperiod.

**Table 1:** Chemical composition of the commercial broiler diet.

Ingredients (%)	Starter diets (1-21d)	Finisher diet (22-35d)
Corn	55.00	57.50
Soybean meal	36.00	34.00
Fish meal	3.00	1.50
Soybean oil	2.00	3.00
Salt	0.35	0.35
Dicalcium phosphate	1.20	1.10
Limestone	1.20	1.00
L-lysine HC1	0.20	0.15
DL-methionine	0.28	0.28
Vitamin premix	0.50	0.50
Mineral premix	0.50	0.50
Total	100	100
Nutrient composition (Calculate)		
CP (%)	22.32	20.75
ME (Mcal/kg)	3.03	3.11
Crude fat/ Ether extract (%)	4.87	5.82
Crude fiber (%)	2.47	2.46
Lysine (%)	1.29	1.17

The premix provided the following per kg of the diets: vitamin A, 18,000 IU; vitamin D3, 2,800 IU; vitamin E, 90 mg; vitamin K3, 7.2 mg; vitamin B1, 6.8 mg; vitamin B2, 27 mg; vitamin B6, 13.5 mg; vitamin B12, 0.1 mg; nicotinamide, 108 mg; calcium pantothenate, 45 mg; folic acid, 19.8 mg; biotin, 0.7 mg; Fe, 100 mg; Cu, 25 mg; Mn, 120 mg; Zn, 80 mg; I, 1 mg; Se, 0.15 mg.

### Growth performance

Body weight of broilers was recorded on days 0, 7, 14, 21, 28, and 35 across all treatment groups. Feed intake was monitored daily throughout the experimental period. Average daily feed intake (**ADFI**) and average daily gain (**ADG**) were calculated based on the recorded feed intake and body weight data. Feed conversion ratio (**FCR**) was determined as the ratio of feed intake to body weight gain.

### Sample collection

On day 35, following a 12-hour fast (with water provided *ad libitum*), 5 birds with body weights representative of the group average were randomly selected from each treatment for sampling. Blood samples were first collected via brachial venipuncture. The chickens were then humanely euthanised via gradual-fill CO_2_ inhalation (30% chamber volume/min), followed by cervical disarticulation. Death was verified by the absence of a pupillary light reflex and heartbeat prior to sampling. Cecal digesta from the 5 selected birds was harvested for 16S rRNA high-throughput sequencing to characterize intestinal microbiota. Jejunal tissues were isolated to quantify the expression of immune and intestinal barrier-related genes. Segments of the duodenum, jejunum and ileum were excised for intestinal morphological measurement. Meanwhile, key immune organs (bursa of Fabricius, thymus and spleen) were carefully separated and weighed to calculate their relative organ weights.

### Serum biochemical and immunoglobulin analysis

Blood samples were centrifuged at 3,000 rpm for 20 min using a Kokusan H-19α centrifuge (Kokusan, Saitama, Japan) to separate the serum. The collected serum was stored at −20°C until further analysis. Serum biochemical parameters were determined using an BioMajesty JCA-BM6010/C according to the manufacturer’s instructions. Serum biochemical parameters determined included total cholesterol (**TC**), triglycerides (**TG**), creatinine (**Cr**), total protein (**TP**), albumin (**ALB**), blood urea nitrogen (**BUN**), aspartate aminotransferase (**AST**), alanine aminotransferase (**ALT**), high-density lipoprotein cholesterol (**HDL-C**), and low-density lipoprotein cholesterol (**LDL-C**). The concentrations of IgA, IgM, and IgY were quantified using commercial enzyme-linked immunosorbent assay (ELISA) kits (Bethyl Laboratories, USA; IgA: cat. no. E33–103, IgM: cat. no. E33–102, IgY/IgG: cat. no. E33–104). Serum samples were diluted appropriately before analysis.

### Relative weights of immune organs

The relative weight index of each immune organ was calculated according to the following formula:

$$\text{Relative}\;\text{organ}\;\text{index}\left(\%\right)=\frac{\text{Weight}\;\text{of}\;\text{immune}\;\text{organ}\left(g\right)}{\text{Live}\;\text{body}\;\text{weight}\;\left(g\right)}\times 100\%$$

where the immune organs referred to the bursa of Fabricius, thymus, and spleen.

### Intestinal morphology

Approximately 2 cm segments were collected from the duodenum, jejunum, and ileum. The Meckel diverticulum was used as the anatomical landmark to identify the jejunoileal junction, with the jejunal sample collected approximately 2 cm proximal and the ileal sample approximately 2 cm distal to this reference point. The samples were thoroughly rinsed with normal saline and subsequently fixed in 10% neutral buffered formalin (Sharifi et al., 2012). Fixed tissues were processed through a graded dehydration series, embedded, and sectioned using a rotary microtome. The sections were stained with hematoxylin and eosin following standard histological procedures (Daneshmand et al., 2017; Habte-Tsion et al., 2015). Morphological measurements, including villus height (**VH**) and crypt depth (**CD**), were obtained at 40 × magnification using a digital imaging system (Motic MC2000) attached to a compound microscope (CX21, Olympus Corporation, Tokyo, Japan). Villus height was defined as the distance from the tip of the villus to the villus–crypt junction, whereas crypt depth was measured as the depth between two adjacent villi (Shini et al., 2020).

### Gene expression

Total RNA was extracted from liver and jejunal tissues using the PureLink RNA Mini Kit (Invitrogen, Thermo Fisher Scientific, USA) according to the manufacturer's protocol, and RNA concentration was determined spectrophotometrically using the NanoDrop 1000 (Thermo Fisher Scientific, USA). Gene-specific primers targeting immune-related genes (tumor necrosis factor-α, interleukin-1β, interleukin-10, and interleukin-12), antioxidant-related genes (superoxide dismutase, catalase, and glutathione peroxidase 1), tight junction and barrier-related genes (mucin 2, claudin 1, occludin, and tight junction-associated protein 1) were designed as listed in Table 2, with β-actin used as the housekeeping gene.

**Table 2:** Primer sequences used for quantitative real-time PCR analysis of antioxidant-related, immune-related genes in broiler jejunum and liver tissues, and intestine barrier-related genes in broiler jejunum tissue.

Target gene	Primer Sequences	Accession No.
*β-actin*	Housekeeping gene	X00182.1
	F: CTGGCACCTAGCACAATGAA	
	R: ACATCTGCTGGAAGGTGGAC	
Antioxidant-related genes		
*SOD*	F: GCCACCTACGTGAACAACCT	
	R: AGTCACGTTTGATGGCTTCC	NM204211
*CAT*	F: CCACGTGGACCTCTTCTTGT	
	R: AAACACTTTCGCCTTGCAGT	
*GPX*	F: CAGCAAGAACCAGACACCAA	NM001031215
	R: CCAGGTTGGTTCTTCTCCAG	GQ502186.2
Immune-related genes		
*IL-10*	F: AGCAGATCAAGGAGACGTTC	NM_001004414.4
	R: ATCAGCAGGGTACTCCTCGAT	
*IL-12*	F: CTGAAGGTTGCAGAAGCAGAG	
	R: CCAGCTCTGCCTTGTAGGTT	NM213588
*IL-1β*	F: GTGAGGCTCAACATTGCGCTGTA	
	R: TGTCCAGGCGGTAGAAGATGAAG	Y15006
*TNF-α*	F: TGCTGTTCTATGACCGCC	
	R: CTTTCAGAGCATCAACGCA	NM204267
Intestine barrier and nutrient transporter-related genes		
*OCLDN*	F: ACGGCAGCACCTACCTCAA	
	R: GGCGAAGAAGCAGATGAG	NM_205128.1
*CLDN1*	F: TGGAGGATGACCAGGTGAAGA	
	R: CGAGCCACTCTGTTGCCATA	NM_001013611.2
*MUC2*	F: ATGCGATGTTAACACAGGACTC	
	R: GTGGAGCACAGCAGACTTTG	JX284122.1
*GLUT2*	F: AGATGACAGCTCGCCTGATG	
	R: GTCTTCAATCACCTTCTGCGG	396130
*TJAP1*	F: AGGAAGCGATGAATCCCTGTT	
	R: TCACTCAGATGCCAGATCCAA	421455

*IL-1β*, interleukin 1 beta; *IL-10*, interleukin 10; *IL-12*, interleukin 12; *TNF-α*, tumor necrosis factor alpha; *SOD*, superoxide dismutase; *CAT*, catalase; *GPX*, glutathione peroxidase; *MUC2*, mucin-2; *CLDN1*, claudin-1; *OCLDN*, occludin; *GLUT2*, glucose transporter 2; *TJAP1*, Tight junction associated protein 1.

Quantitative real-time PCR (qPCR) was performed using the qPCRBIO SyGreen 1-Step Go Lo-ROX kit (PCR Biosystems, London, UK) on a StepOnePlus Real-Time PCR System. Each reaction was conducted in a final volume of 20 μL, containing 10 μL of 2× qPCRBIO SyGreen 1-Step Mix, 0.8 μL of 10 μM forward primer, 0.8 μL of 10 μM reverse primer, 1 μL of 20× RTase Go, 10 ng of total RNA, and nuclease-free water to volume. The thermal cycling conditions consisted of an initial hold at 42°C for 2 minutes, reverse transcription at 45°C for 10 minutes, polymerase activation at 95°C for 2 minutes, followed by 40 cycles of denaturation at 95°C for 5 seconds and annealing/extension at 65°C for 30 seconds. A melt curve analysis was subsequently performed to confirm amplification specificity. Relative gene expression levels were calculated using the 2^−ΔΔCt^ method in accordance (Larionov et al., 2005).

### Microbiota analysis and bioinformatics

Total DNA was extracted from the cecal contents of broilers in the Control and GLE-supplemented groups (GLE125, GLE250, and GLE500) using the ZymoBIOMICS®-96 MagBead DNA Kit (Zymo Research, Irvine, CA) according to the manufacturer’s instructions. The concentration and purity of the extracted DNA were assessed via spectrophotometry (Nanodrop ND1000; Thermo Scientific, USA). The V3-V4 regions of the bacterial 16S ribosomal RNA genes were amplified through polymerase chain reaction (PCR) using the Quick-16S™ NGS Library Prep Kit (Zymo Research, Irvine, CA) with the universal primers 338F (5′-ACTCCTACGGGAGGCAGCAG-3′) and 806R (5′-GGACTACHVGGGTWTCTAAT-3′). Real-time PCR was performed during library preparation to ensure optimal amplification cycles. The final PCR products were quantified using quantitative PCR (qPCR), pooled based on their molar concentrations, and cleaned using the Select-a-Size DNA Clean & Concentrator™ (Zymo Research, Irvine, CA). The final library quality was validated using TapeStation® (Agilent Technologies, USA) and Qubit® (Thermo Fisher Scientific, USA) before sequencing on the Illumina platform.

Raw reads were processed using the DADA2 pipeline within QIIME v.1.9.1. Following the removal of sequencing errors, low-quality reads, and chimeras, unique amplicon sequence variants (ASVs) were identified(Callahan et al., 2016). Taxonomic classification was performed using Uclust in QIIME v.1.9.1 with the Zymo Research 16S database as the reference. The α-diversity indices, including the Simpson diversity index and Observed species (Sobs), were calculated to characterize microbial α-diversity. Beta diversity was assessed based on Bray-Curtis dissimilarity to evaluate overall differences in microbial community structure.

### Statistical analysis

All experimental data were analyzed using analysis of variance (ANOVA) with SPSS Statistics (IBM SPSS Inc., Chicago, IL, USA) and SAS Enterprise Guide (SAS Institute, Cary, NC, USA). Differences among treatment groups were evaluated using Duncan’s multiple range test. Statistical significance was declared at *P* < 0.05. Significant differences in microbial community structure among the 4 experimental groups were statistically determined using permutational multivariate analysis of variance (PERMANOVA).

## Results

### Chemical properties of GLE

Garlic leaves were extracted via maceration in 85% (v/v) ethanol to yield the bioactive crude extract. The chemical characteristics of this ethanolic extract were subsequently analysed and are summarized in Table 3. The total phenolic content was determined to be 4.62 ± 0.23 mg of GAE per gram of crude extract, and the total flavonoid content was 1.71 ± 0.33 mg of CE per gram. Furthermore, specific bioactive polyphenols were quantified, notably identifying caffeic acid (0.96 ± 0.07 mg/g), gallic acid (0.58 ± 0.06 mg/g), and catechin (0.34 ± 0.05 mg/g). The remaining seven targeted polyphenols, mangiferin, epicatechin, rosmarinic acid, quercetin, lutein, vanillic acid, and p-coumaric acid, were non-detectable within the concentration range of the calibration standards used (0 to 100 ppm). The antioxidant capacity of the extract was evaluated using DPPH and ABTS radical scavenging assays. The extract exhibited a DPPH scavenging activity of 9.84 ± 1.18 mg of Trolox Equivalents (TE) per gram of crude extract, corresponding to a high decolourisation rate of 89.00 ± 0.73% at a concentration of 50 mg/mL. The garlic leaf extract demonstrated a potent ABTS scavenging capacity of 115.17 ± 3.69 mg TE/g of crude extract, achieving an outstanding scavenging activity rate of 247.10 ± 1.09% at a concentration of 1 mg/mL.

**Table 3:** The chemical properties of garlic leave extracts.

Chemical properties	Value
Reducing sugar (mg/g)	118.39±6.40
Total phenolic content (mgGAe/g)	4.62±0.23
Total Flavonoid content (mgCE/g)	1.71±0.33
Gallic acid (mg/g)	0.58±0.06
Mangiferin (mg/g)	Nd
Catechin (mg/g)	0.34±0.05
Epicatechin (mg/g)	Nd
Caffeic acid (mg/g)	0.96±0.07
Rosmarinic acid (mg/g)	Nd
Quercetin (mg/g)	Nd
Lutein (mg/g)	Nd
Vanillic acid (mg/g)	Nd
p-coumaric acid (mg/g)	Nd
*DPPH scavenging activity (mgTE/g)	9.84±1.18
*DPPH scavenging activity (%)	89.00±0.73
*ABTS scavenging activity (mgTE/g)	115.17±3.69
*ABTS scavenging activity (%)	247.10±1.09

Values are mean ± SE per gram of ethanolic crude extract. nd is not detectable within the range of standard used.

⁎ The scavenging activity values were showed at the concentration of crude extract at 100 mg/mL.

### Growth performance

The impact of GLE supplementation on broiler performance throughout the 35-day experimental period is presented in Table 4. There was no significant difference (*P* > 0.05) in ADFI among the treatments during each period. Final body weight and ADG were significantly (*P*<0.05) increased in the GLE125 group compared to Control and GLE250 at 35 days. Furthermore, broilers administered GLE 125 exhibited a reduced FCR (*P* < 0.05).

**Table 4:** Effects of garlic leave extract on growth performance of broiler chickens at 35 days of experiment.

Items	Control	GLE125	GLE250	GLE500	SEM	*P*-value
Initial body weight	42.7	42.8	43.2	42.8	0.417	0.8363
Final body weight	1966.7^b^	2143.3^a^	1990.0^b^	2055.0^ab^	29.464	0.0116
ADG; g/day	55.0^b^	60.0^a^	55.6^b^	57.5^ab^	0.851	0.0124
ADFI; g/hen/day	81.5	80.7	81.0	80.2	0.485	0.3282
FCR	1.5^a^	1.3^c^	1.5^ab^	1.4^bc^	0.019	0.0039

Abbreviations: ADG, Average Daily Gain; ADFI, Average daily feed intake; Feed conversion ratio, FCR; Control, basal diet supplemented with GLE 0 mg/kg diet; GLE 125, basal diet supplemented with GLE 125 mg/kg diet; GLE 250, basal diet supplemented with GLE 250 mg/kg diet; GLE 500, basal diet supplemented with GLE 500 mg/kg diet.

a,b Means within a row with different superscripts differ significantly (*P* < 0.05).

### Serum biochemical profiles

Table 5 presents the effects of GLE supplementation on blood biochemical parameters in broilers at 35 days of age. Statistical analysis indicated that the supplementation groups did not significantly influence (*P* > 0.05) any of the measured parameters, including total cholesterol, triglycerides, total protein, albumin, globulin, glucose, creatin, high-density lipoprotein cholesterol (HDL-C), and low-density lipoprotein cholesterol (LDL-C), aspartate aminotransferase (AST), and alanine aminotransferase (ALT). These results suggest that GLE supplementation had no adverse effects on blood biochemistry, and all measured parameters remained within normal physiological ranges.

**Table 5:** Effects of garlic leave extract on blood biochemistry of broiler chickens at 35 days of experiment.

Variables	Control	GLE125	GLE250	GLE500	SEM	*P*-value
Glucose (mg/dL)	163.3	213.4	177.0	184.5	14.544	0.057
BUN (mg/dL)	2.2	2.2	2.4	2.2	0.265	0.933
Creatin (mg/dL)	0.1	0.1	0.1	0.1	0.012	0.830
AST (U/L)	272.4	269.8	241.2	242.2	10.025	0.068
ALT (U/L)	1.8	1.8	1.4	1.6	0.224	0.547
Total protein (mg/dL)	2.7	2.7	2.7	2.7	0.115	0.993
Albumin (mg/dL)	1.2	1.2	1.3	1.2	0.060	0.704
Globulin (mg/dL)	1.5	1.4	1.4	1.5	0.089	0.730
Total cholesterol (mg/dL)	95.4	104.2	101.2	95.8	3.546	0.263
Triglycerides (mg/dL)	24.8	27.0	26.0	29.8	4.080	0.844
HDL-C (mg/dL)	67.6	77.2	78.4	72.0	3.235	0.109
LDL-C (mg/dL)	23.0	21.6	17.4	17.8	2.256	0.249

Abbreviations: BUN, blood urea nitrogen; AST, aspartate-aminotransferase; ALT, alanine-aminotransferase; high-density lipoprotein cholesterol (HDL-C), and low-density lipoprotein cholesterol (LDL-C), Control, basal diet supplemented with GLE 0 mg/kg diet; GLE 125, basal diet supplemented with GLE 125 mg/kg diet; GLE 250, basal diet supplemented with GLE 250 mg/kg diet; GLE500, basal diet supplemented with GLE 500 mg/kg diet.

### Intestinal morphology

The effects of GLE on the intestinal morphology of broiler chickens are presented in Table 6. GLE supplementation significantly increased VH in both the jejunum and ileum compared with the control group (*P* = 0.0032 and *P* < 0.0001, respectively). However, no significant differences (*P* > 0.05) were observed in CD or the villus height-to-crypt depth ratio (VH:CD) among the treatment groups.

**Table 6:** Effects of garlic leave extract on intestinal morphology of broiler chickens.

Variables	Control	GLE125	GLE250	GLE500	SEM	*P*-value
Duodenum						
VH (μm)	1336.7	1483.3	1336.7	1366.7	48.618	0.1377
CD (μm)	239.2	270.5	242.5	256.7	12.258	0.2797
VH:CD	5.7	5.5	5.6	5.3	0.302	0.8387
Jejunum						
VH (μm)	1190.0^b^	1313.3^a^	1315.0^a^	1305.0^a^	23.990	0.0032
CD (μm)	219.2	262.5	235.7	240.8	10.205	0.0513
VH:CD	5.5	5.0	5.6	5.5	0.227	0.3273
Ileum						
VH (μm)	961.7^c^	1135.0^a^	1156.7^a^	1060.0^b^	21.484	<.0001
CD (μm)	213.83	212.50	235.83	218.17	7.812	0.1616
VH:CD	4.70	5.36	4.91	4.89	0.191	0.0587

Abbreviations: VH, Villus height; CD, Crypt depth; VH:CD, the ratio of villus height to crypt depth. Control, basal diet supplemented with GLE 0 mg/kg diet; GLE 125, basal diet supplemented with GLE 125 mg/kg diet; GLE 250, basal diet supplemented with GLE 250 mg/kg diet; GLE 500, basal diet supplemented with GLE 500 mg/kg diet.

a,b Means within a row with different superscripts differ significantly (*P* < 0.05).

### Relative weights of immune organs

The effects of GLE supplementation on the relative weights of immune organs in broiler chickens are presented in Table 7. Supplementation had no significant effect (*P* > 0.05) on the relative weights of the spleen and thymus. However, Dietary inclusion of GLE significantly increased the relative weight of the bursa of Fabricius (*P* < 0.05), with GLE125 and GLE500 showing significant differences, while GLE250 did not differ significantly from the control.

**Table 7:** Effects of dietary garlic leave extract supplementation on relative weights of immune organs of broiler chickens.

Variables	Control	GLE125	GLE250	GLE500	SEM	*P*-value
Bursa (%)	0.13^b^	0.17^a^	0.15^ab^	0.16^a^	0.009	0.044
Spleen (%)	0.09	0.09	0.08	0.07	0.009	0.498
Thymus (%)	0.16	0.16	0.15	0.15	0.021	0.995

Abbreviations: Control, basal diet supplemented with GLE 0 mg/kg diet; GLE 125, basal diet supplemented with GLE 125 mg/kg diet; GLE 250, basal diet supplemented with GLE 250 mg/kg diet; GLE 500, basal diet supplemented with GLE 500 mg/kg diet.

a,b Means within a row with different superscripts differ significantly (*P* < 0.05).

### Serum immunoglobulin

**Fig. 1:**
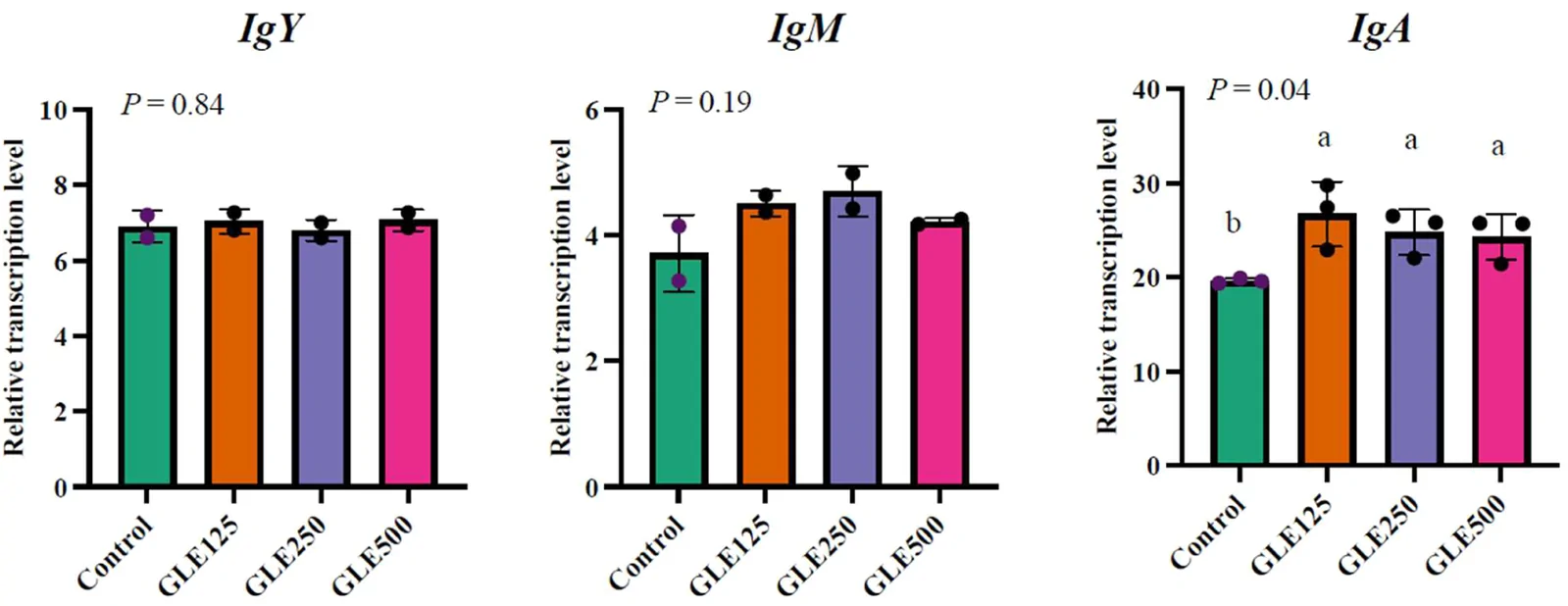
Effects of dietary GPE supplementation on serum immunoglobulin concentrations (IgY, IgM, and IgA) in broiler chickens. Control, basal diet supplemented with GLE 0 mg/kg diet; GLE 125, basal diet supplemented with GLE 125 mg/kg diet; GLE E250, basal diet supplemented with GLE E 250 mg/kg diet; GLE E500, basal diet supplemented with GLE E 500 mg/kg diet. Data are presented as mean ± standard error. Different letters indicate significant differences among groups (*P*< 0.05).

The effects of GLE supplementation on serum immunoglobulin levels in broiler chickens are presented in Fig. 1. The result indicates that the GLE125 treatment group exhibited the highest serum IgA concentration, which was significantly greater than that of the other experimental groups. In contrast, no significant differences were observed in IgM and IgY concentrations among the treatments.

### Cecal microbial community structure

**Fig. 2:**
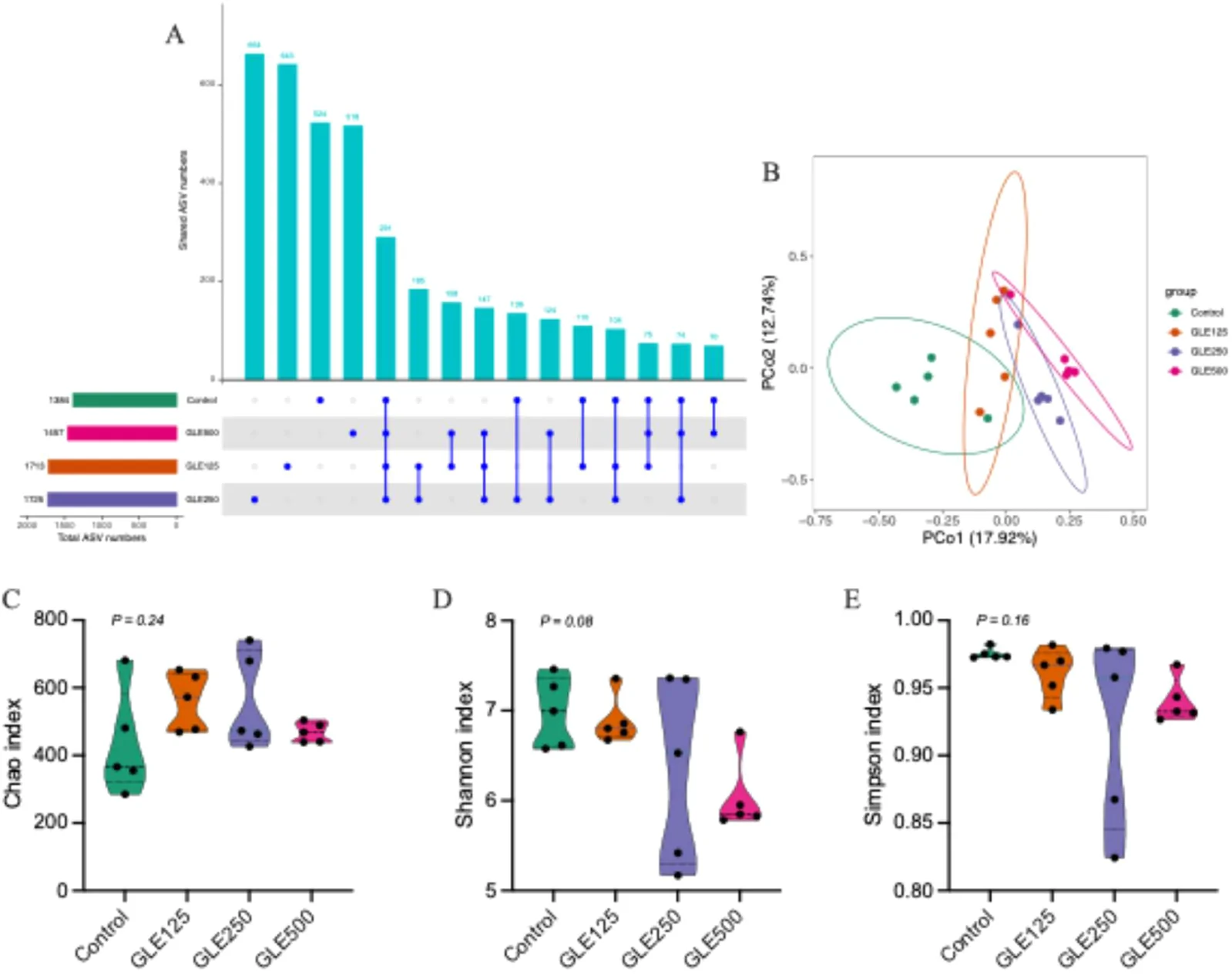
Cecal microbial community structure and diversity indices of broilers supplemented with different levels of garlic leaf extract (GLE). (A) UpSet diagram illustrating the shared and unique operational taxonomic units (OTUs) among the Control, GLE125, GLE250, and GLE500 groups. (B) Principal Coordinate Analysis (PCoA) based on Bray-Curtis dissimilarity, showing the clustering of microbial communities (n = 5 per group). (C) Chao index, (D) Shannon index and (E) Simpson index representing the α-diversity of the cecal microbiota. Data are presented as mean ± SEM. Statistical significance was determined by PERMANOVA for PCoA and one-way ANOVA for α-diversity (*P*> 0.05).

Total DNA was extracted from the cecal samples of the 4 experimental groups (Control, GLE125, GLE250, and GLE500). A total of 291 amplicon operational taxonomic units (OTUs) were shared among all groups. Fig. 2A presents an UpSet diagram illustrating the OTUs shared and unique to each treatment. In addition to the core microbial community, each group possessed a distinct set of unique OTUs: 524 in the Control, 643 in the GLE125, 664 in the GLE250, and 518 in the GLE500 group, indicating that GLE supplementation at different doses exerted treatment-specific effects on the gut microbial repertoire.

The α-diversity of the cecal microbiota was evaluated using the Chao (Fig. 2C), Shannon (Fig. 2D) and Simpson (Fig. 2E) indices. The results indicated that no statistically significant differences in microbial richness or evenness were detected among the experimental groups (*P* > 0.05), suggesting that GLE supplementation maintained the overall ecological stability of the cecal microbiota. However, the dissimilarity in bacterial community structure was examined through PCoA based on Bray-Curtis dissimilarity (Fig. 2B). The difference among the 4 treatments was determined by PERMANOVA, which revealed a clear separation of microbial communities along the PCoA1 and PCoA2 axes. These two axes explained 17.92% and 12.74% of the total variation, respectively, suggesting that GLE supplementation significantly reshaped the microbial community structure despite the lack of change in α-diversity.

### Discriminatory cecal microbial taxa

**Fig. 3:**
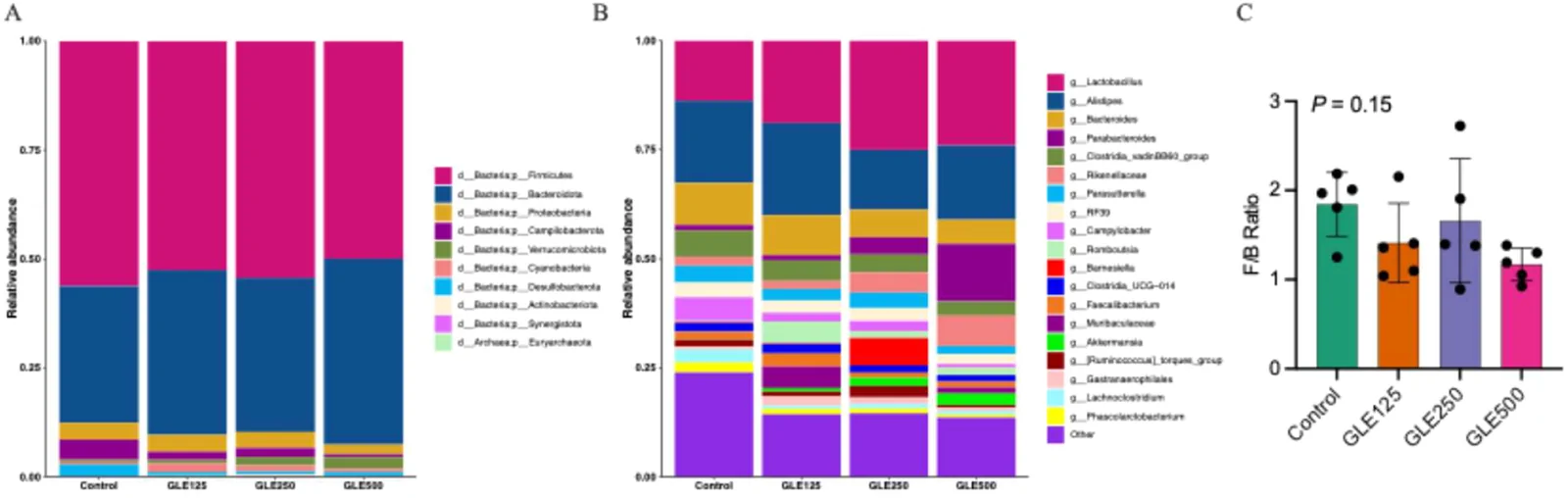
Relative abundance of cecal microbiota at the phylum and genus levels. (A) Composition of the top 10 bacterial phyla across the 4 experimental groups. (B) Relative abundance of the top 20 bacterial genera. (C) The ratio of Firmicutes to Bacteroidetes (F/B ratio) in the cecal contents. Each bar represents the average relative abundance within the respective treatment group. Data were analyzed using one-way ANOVA (*P*< 0.05).

The relative abundance of the predominant bacterial top10 phyla and top20 genera in the ceca is shown in Fig. 3. At the phylum level, *Firmicutes* and *Bacteroidetes* were the dominant phyla across all groups, collectively accounting for the vast majority of the identified sequences (Fig. 3A). At the genus level, the predominant taxa across all treatments included *g__Lactobacillus, g__Alistipes, g__Bacteroides* and *g__Parabacteroides* (Fig. 3B). The Firmicutes-to-Bacteroidetes (F/B) ratio showed fluctuations among groups, with a visible shift in the GLE-supplemented groups compared to the Control, although the magnitude of change varied with the GLE inclusion level (Fig. 3C).

**Fig. 4:**
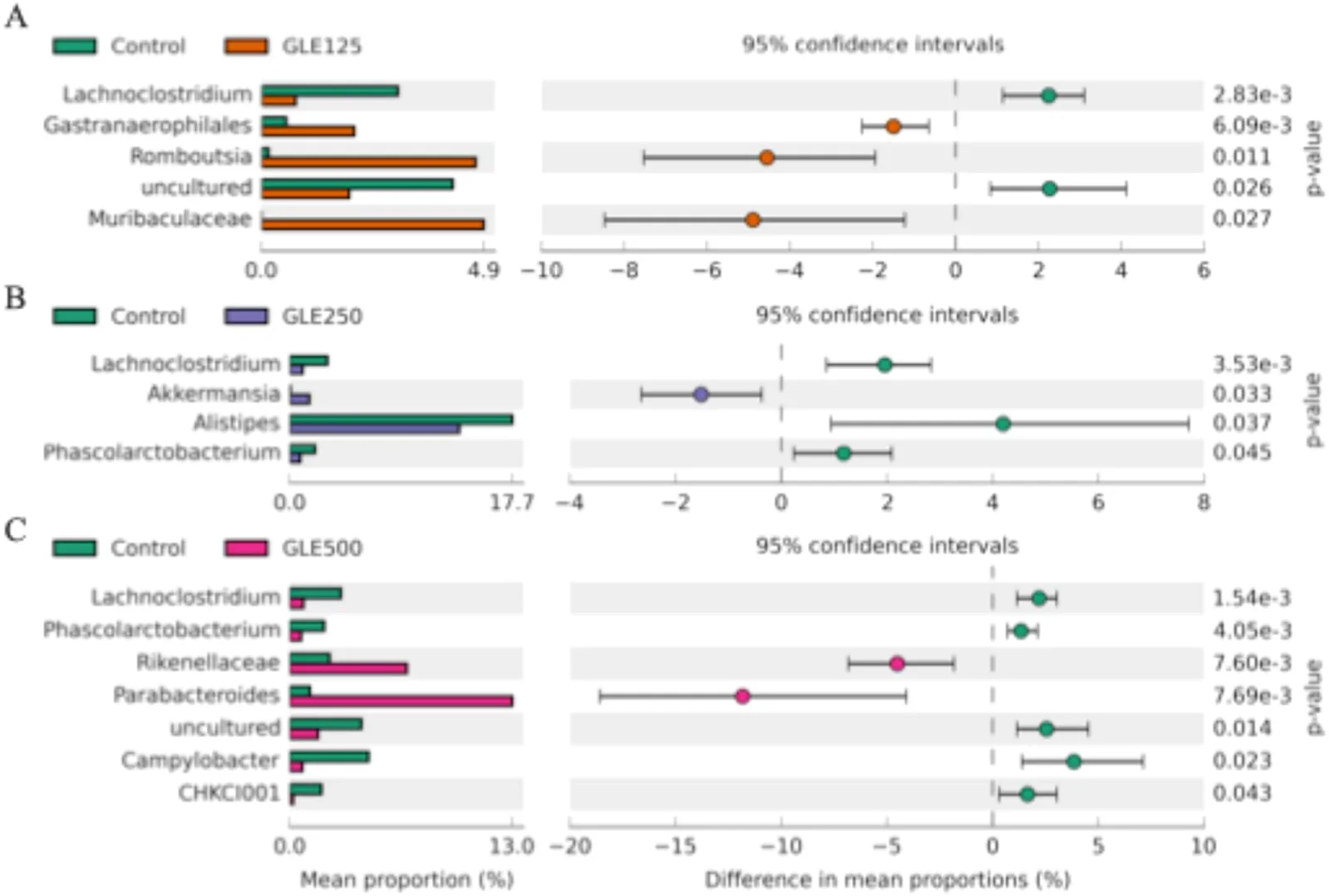
Effects of different levels of garlic leaf extract (GLE) on differentially abundant cecal bacterial genera in broiler chickens. Genera with significant differences were identified using STAMP based on pairwise comparisons: (A) control and GLE125 groups, (B) control and GLE250 groups, and (C) control and GLE500 groups. Statistical significance was determined using Welch’s t test (*P*< 0.05).

Pairwise comparisons using STAMP demonstrated that the genera *Lachnoclostridium* was significantly altered in GLE groups compared with the control group (*P* < 0.05). In addition, *Gastranaerophilales, Romboutsia*, and *Muribaculaceae* genera significantly affected by GLE125 supplementation are shown in Fig. 4A, whereas *Akkermansia* genera significantly influenced by GLE250 supplementation are presented in Fig. 4B. Furthermore, significant differences in the relative abundance of *Rikenellaceae*, and *Parabacteroides* were observed between the Control and GLE500 groups (Fig. 4C).

### Immune and antioxidant-related gene expression in liver

**Fig. 5:**
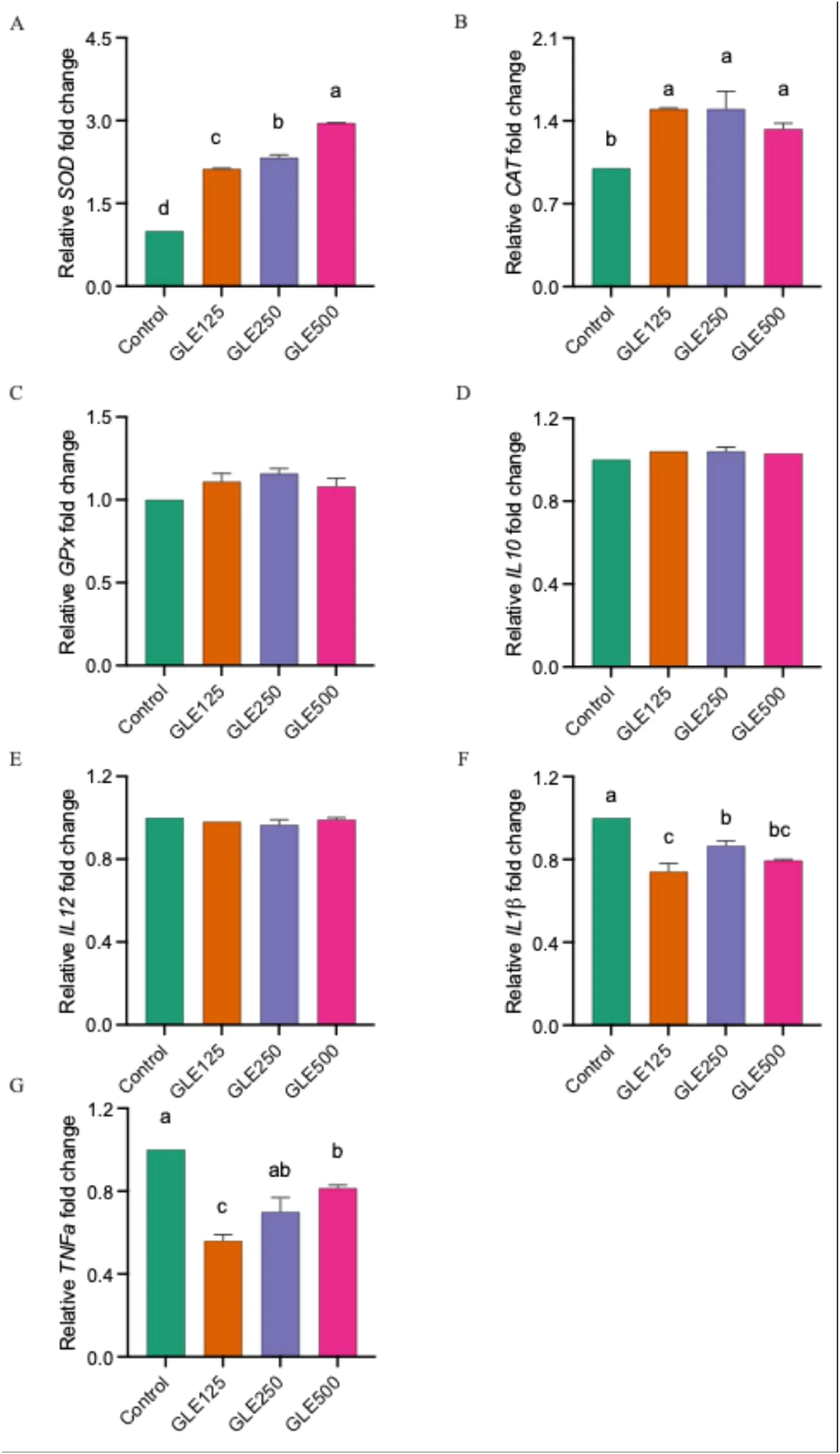
Relative mRNA expression of antioxidant- and immune-related genes (*SOD, CAT, GPx, IL-10, IL-1β, IL12, TNF-α*) in liver tissue of broiler chickens at 35 days. Control, basal diet supplemented with GLE 0 mg/kg diet; GLE 125, basal diet supplemented with GLE 125 mg/kg diet; GLE 250, basal diet supplemented with GLE 250 mg/kg diet; GLE 500, basal diet supplemented with GLE 500 mg/kg diet. Data are presented as mean ± standard error. Different letters indicate significant differences among groups (*P*< 0.05).

To further evaluate the impact of garlic leaf extract on the hepatic health of broiler chickens, the mRNA basal expression levels of key inflammatory signaling molecules and antioxidant enzymes were determined (Fig. 5). As shown in Fig. 5, GLE treatment significantly upregulated the expression of hepatic antioxidant genes *SOD* and *CAT* (*P* < 0.05), though their induction patterns differed. *SOD* expression was increased in a dose-dependent manner with rising GLE concentrations, and statistically significant differences were observed between all groups. In contrast, *CAT* expression was significantly elevated following low-dose GLE treatment and remained stable across the 125–500 concentration range, with no further dose-dependent effect. No significant differences in *GPX* expression were detected between the control and all GLE-treated groups. GLE exerted selective regulatory effects on the expression of pro-inflammatory cytokine genes. The expression levels of *IL-12* and *IL-10* were comparable between the control and all GLE-treated groups. However, the expression of *IL-1β* and *TNF-α* was significantly suppressed by GLE, both exhibiting a "decrease followed by partial recovery" pattern: low-dose GLE (GLE125) treatment reduced *IL-1β* and *TNF-α* expression to their lowest levels. Although expression levels partially recovered at medium and high doses, they remained significantly lower than those in the control group.

### Immune and antioxidant-related gene expression in Jejunum

**Fig. 6:**
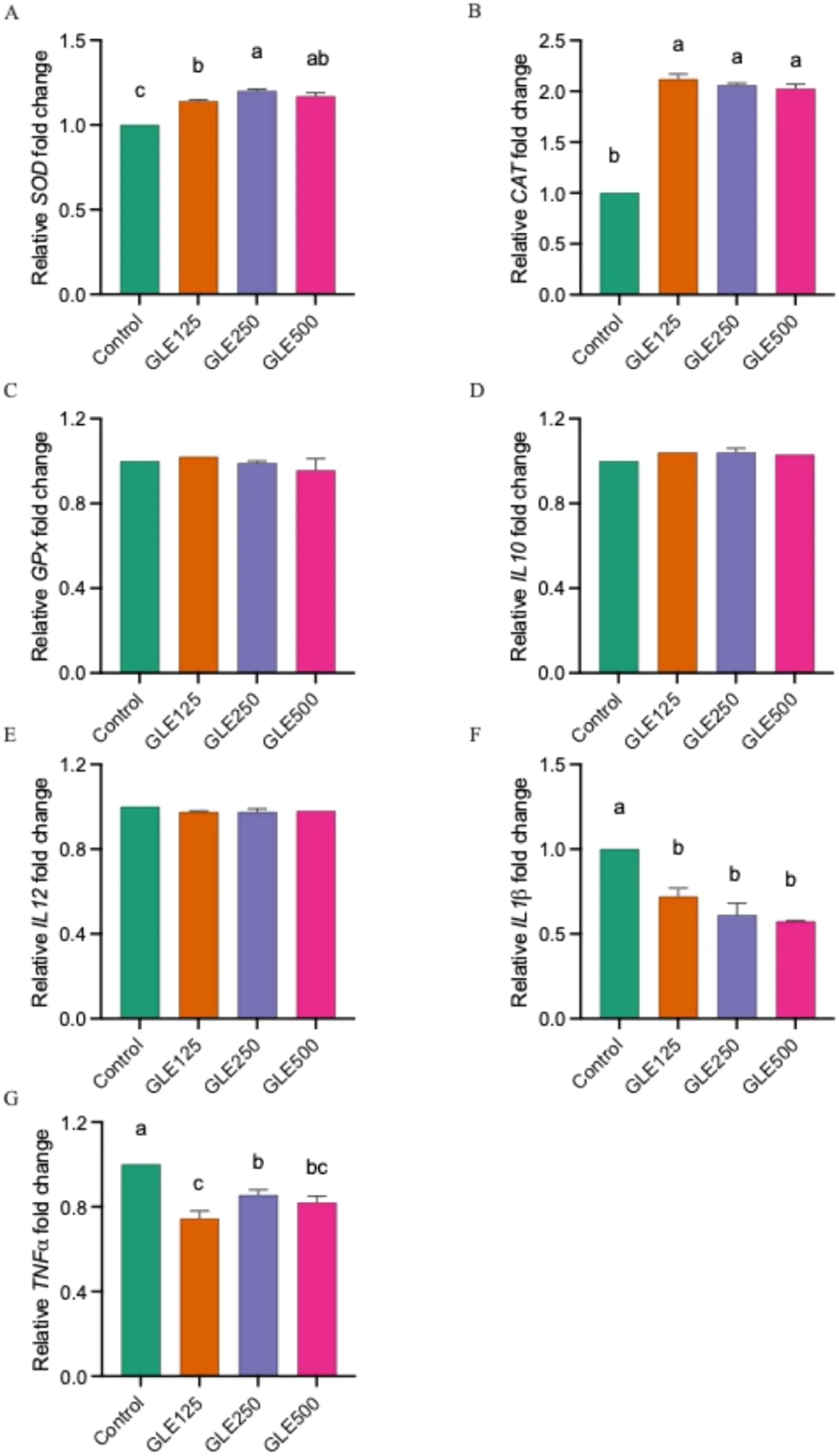
Relative mRNA expression of antioxidant- and immune-related genes (*SOD, CAT, GPx, IL-10, IL-1β, IL12, TNF-α*) in jejunum tissue of broiler chickens at 35 days. Control, basal diet supplemented with GLE 0 mg/kg diet; GLE 125, basal diet supplemented with GLE 125 mg/kg diet; GLE 250, basal diet splemented with GLE 250 mg/kg diet; GLE 500, basal diet supplemented with GLE 500 mg/kg diet. Data are presented as mean ± standard error. Different letters indicate significant differences among groups (*P*< 0.05).

To explore the regulatory effects of GLE on intestinal oxidative stress and inflammation, the expression of key genes in the jejunum was analysed (Fig. 6). The results showed that GLE activated the jejunal antioxidant system through distinct patterns. The induction of *SOD* exhibited a clear dose-response relationship, with expression continuously increasing with concentration and peaking at 250 μg/mL. The induction of *CAT* showed a low-concentration sensitivity pattern, with significant upregulation observed at 125 μg/mL, and no further enhancement with higher concentrations. No regulatory effect of GLE on *GPX* expression was detected, suggesting it is not a major target of jejunal antioxidant response.

In terms of inflammatory regulation, GLE mainly targeted the pro-inflammatory factors *IL-1β* and *TNF-α*. The *IL-1β* maintained low expression levels in all GLE-treated groups with no significant concentration difference. The inhibitory effect on *TNF-α* decreased with increasing concentration, with the strongest inhibition observed at low-dose GLE. Although partial recovery occurred at medium and high doses, expression levels remained significantly lower than those in the control group.

### Expression of intestinal barrier function and nutrient transporter-related genes in the jejunum

**Fig. 7:**
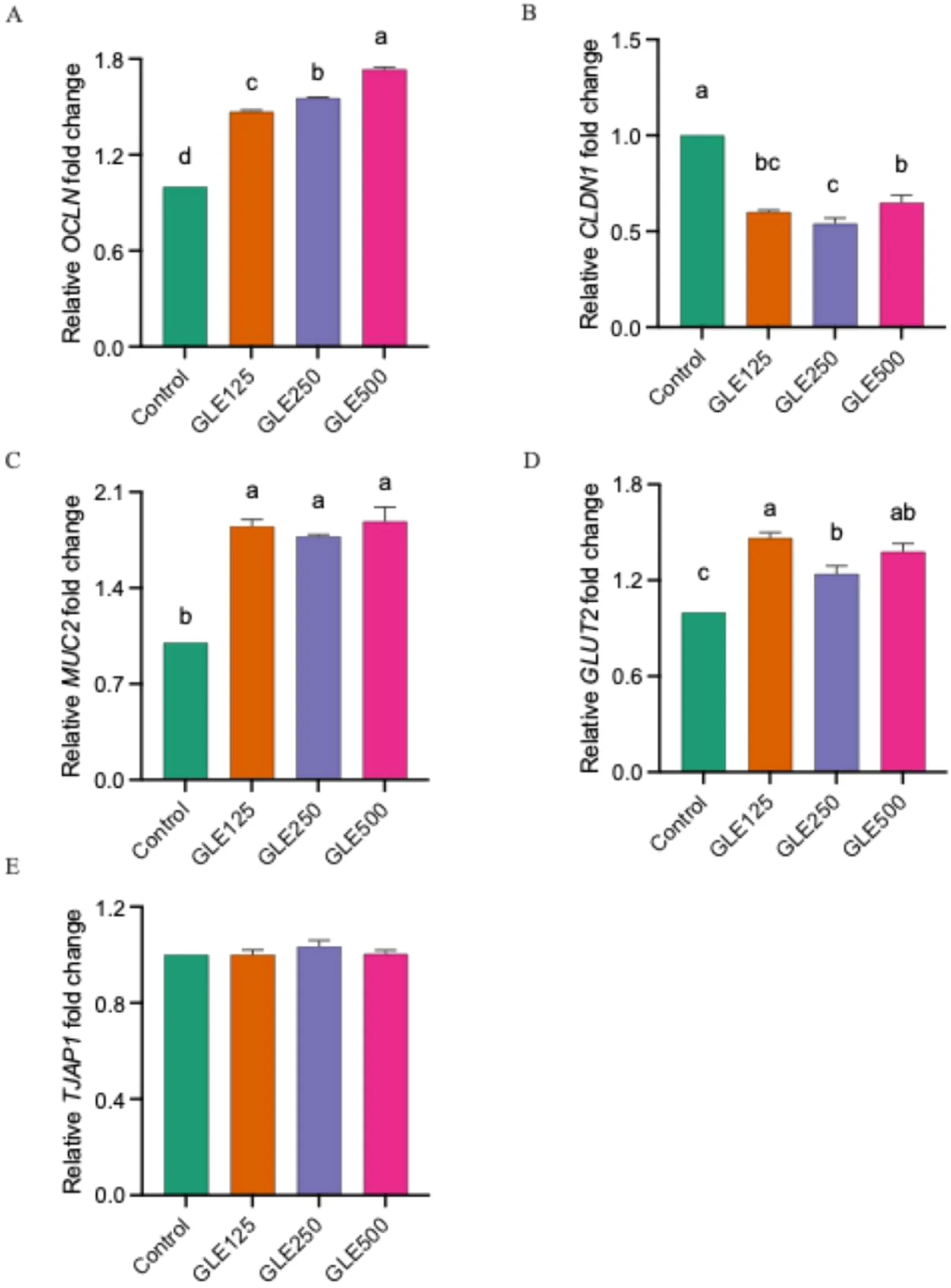
Relative mRNA expression of intestine barrier and nutrient transporter-related genes (*OCLDN, CLDN1, TJAP1, MUC2, GLUT2*) in jejunum tissue of broiler chickens at 35 days. Control, basal diet supplemented with GLE 0 mg/kg diet; GLE 125, basal diet supplemented with GLE 125 mg/kg diet; GLE 250, basal diet supplemented with GLE 250 mg/kg diet; GLE 500, basal diet supplemented with GLE 500 mg/kg diet. Data are presented as mean ± standard error. Different letters indicate significant differences among groups (*P*< 0.05).

The expression of genes related to jejunal barrier function and nutrient transport was further assessed, as shown in Fig. 7. For tight junction genes, *OCLN* expression increased dose-dependently, peaking in the GLE500 group (*P* < 0.05). In contrast, *CLDN1* was significantly suppressed, showing a "decrease-then-recovery" pattern with its nadir at GLE250 (*P* < 0.05). No significant changes were observed for *TJAP1* across all groups (*P* > 0.05).

For the mucosal protective gene *MUC2*, GLE treatment induced a plateau-like up-regulation; all GLE-supplemented groups exhibited significantly higher transcript levels than the control (*P* < 0.05), with no inter-dose differences. Similarly, *GLUT2* expression was significantly up-regulated (*P* < 0.05), most notably in the GLE125 group, although all treatment levels remained elevated relative to the control. Overall, GLE improves jejunal barrier function via *OCLN* and *MUC2* induction and potentially enhances nutrient absorption through up-regulated *GLUT2* expression.

## Discussion

A primary mechanism driving the enhanced growth performance in garlic-supplemented broilers is the improvement of the intestinal microenvironment. Garlic biomass contains a substantial amount of non-structural carbohydrates, specifically water-soluble fructans, fructo-oligosaccharides (FOS), and reducing sugars (Kothari et al., 2019; Sunanta et al., 2023a). When incorporated into poultry feed, these complex sugars escape upper gastrointestinal digestion and act as potent prebiotics in the hindgut. They provide fermentable substrates that selectively stimulate the proliferation of beneficial commensal bacteria, such as *Lactobacillus* and *Bifidobacterium* species, while competitively excluding pathogenic microbes like *Escherichia coli, Salmonella,* and *Clostridium perfringens* (Chen et al., 2021). This microbial shift, often referred to as gut modulation, actively prevents intestinal dysbiosis and fortifies the gut barrier. Furthermore, the fermentation of these prebiotics promotes optimal intestinal histomorphology, significantly increasing villus height, villus width, and crypt depth (Chen et al., 2021; Kothari et al., 2019). Larger intestinal villi vastly increase the absorptive surface area of the gut, thereby enhancing the apparent total tract retention of nutrients and ultimately maximising the FCR (Kothari et al., 2019). Beyond its carbohydrate profile, garlic is an abundant source of vital secondary metabolites, particularly phenolics and flavonoids. Extracts from garlic, especially the leaf biomass, yield high concentrations of polyphenols such as caffeic acid, gallic acid, and catechin (Sunanta et al., 2020; Sunanta et al., 2023a). Because these compounds possess an aromatic ring with hydroxyl groups, they function as highly efficient electron donors that neutralise free radicals, conferring exceptional antioxidant capacity as demonstrated by DPPH and ABTS scavenging assays (Somamno et al., 2016; Sunanta et al., 2020).

In fast-growing commercial broilers, oxidative stress can severely impair cellular health and metabolic efficiency. The dietary inclusion of these antioxidant-rich natural additives prevents lipid peroxidation and structural damage to the intestinal epithelium (Chen et al., 2021; Sunanta et al., 2023b). Furthermore, these antioxidant compounds interact directly with the host's immune pathways. Studies have demonstrated that garlic supplementation bolsters the innate immune response by enhancing phagocytosis, increasing white blood cell, basophil, and monocyte counts, and downregulating pro-inflammatory cytokines (Elagib et al., 2013; Kothari et al., 2019; Mufungwe et al., 2024). By mitigating oxidative and immunological stress, the bird diverts less metabolic energy toward disease resistance and tissue repair, reallocating it toward muscle accretion and growth(Klasing, 2007).

The combined prebiotic action of garlic's sugar content and the protective effects of its phenolic and flavonoid antioxidants culminate in superior overall flock performance. Numerous in vivo trials corroborate that dietary garlic supplementation at optimised levels (e.g., between 0.3% to 3.0%) stimulates appetite and gastric juice secretion, leading to increased feed intake, higher final body weight, and significantly improved daily body weight gain (Ari et al., 2012; Elagib et al., 2013; ELTAZI et al., 2014; Mufungwe et al., 2024). Additionally, the hypocholesterolemic effects of these bioactive compounds lead to improved lipid metabolism, producing higher quality, leaner meat with reduced abdominal fat deposition and lower serum cholesterol (Ari et al., 2012; ELTAZI et al., 2014). The present study demonstrated that dietary supplementation with GLE, particularly at 125 mg/kg, significantly improved final body weight, ADG, and FCR in broiler chickens, without affecting ADFI. The unchanged ADFI across treatments suggests that the observed improvements in growth performance were not driven by increased feed consumption but rather by enhanced feed utilisation efficiency. This finding is consistent with previous reports indicating that phytogenic feed additives derived from garlic can improve nutrient digestibility and metabolic efficiency in poultry (Amad et al., 2011; Toghyani et al., 2011).

The improvement in ADG and FCR observed in the GLE125 group may be attributed to the bioactive compounds present in garlic leaves, such as organosulfur compounds, flavonoids, and phenolic constituents, which have been shown to possess antimicrobial and digestive-stimulatory properties. These compounds can modulate gut microbiota, suppress pathogenic bacteria, and enhance the activity of digestive enzymes, thereby improving nutrient absorption and growth performance(Windisch et al., 2008) (Windisch et al., 2008; Rehman et al., 2018). In line with this, garlic-derived additives have been widely reported to enhance broiler performance by improving gut health and intestinal integrity. Interestingly, the beneficial effects were most pronounced at the 125 mg/kg inclusion level, while higher doses did not yield further improvements. This dose-dependent response may indicate that moderate supplementation levels optimise physiological responses, whereas excessive inclusion could lead to reduced efficacy or metabolic burden. Similar observations have been reported in studies where optimal levels of phytogenic additives produced maximal growth benefits, beyond which no additional improvements were observed(García et al., 2007). Moreover, the improvement in FCR suggests that GLE supplementation enhanced feed efficiency, likely through improved intestinal morphology and nutrient transporter activity, as supported by previous findings linking gut structural development with better feed utilisation (Awad et al., 2009). The ability of GLE to improve growth performance without increasing feed intake is particularly advantageous from a production and economic perspective.

The present study demonstrated that GLE supplementation did not significantly alter serum biochemical parameters in broilers, indicating that GLE has no adverse effects on liver function, lipid metabolism, or overall physiological status. The stability of markers such as AST, ALT, cholesterol, and glucose suggests that GLE is safe for dietary inclusion, which is consistent with previous studies reporting that garlic-derived additives do not negatively influence blood biochemical profiles in poultry (Khan et al., 2012; Toghyani et al., 2011). This finding supports the use of phytogenic additives as safe alternatives to AGPs. In terms of intestinal morphology, GLE supplementation significantly increased VH in the jejunum and ileum, suggesting enhanced absorptive capacity of the intestine. Increased VH is commonly associated with improved nutrient digestion and absorption, which may contribute to better growth performance. Similar improvements in intestinal structure have been reported with the use of phytogenic compounds, including garlic, which can stimulate epithelial cell proliferation and reduce intestinal inflammation (Awad et al., 2009; Reisinger et al., 2011). The lack of change in CD and VH:CD ratio indicates that GLE primarily promoted mucosal development without inducing excessive tissue turnover.

Regarding immune organ development, the significant increase in bursal weight observed in GLE-supplemented groups suggests a positive effect on humoral immune function, as the bursa of Fabricius plays a critical role in B-cell maturation in birds. Previous studies have shown that phytogenic feed additives, particularly those derived from garlic, can enhance immune organ development and stimulate lymphoid tissue activity (Wang et al., 2024). In terms of innate immunity, GLE supplementation significantly upregulated *SOD* and *CAT* expression in the liver and jejunum while selectively suppressing pro-inflammatory cytokines *IL-1β* and *TNF-α* without affecting *IL-10* and *IL-12*, indicating modulation of the innate immune response through antioxidant and anti-inflammatory pathways. Regarding adaptive immunity, the elevated serum IgA concentration in the GLE125 group reflects stimulation of humoral immunity; IgA produced by B cells in gut-associated lymphoid tissue serves as a bridge between innate and adaptive systems, providing specific pathogen neutralization while maintaining mucosal homeostasis. While serum IgA is predominantly monomeric and derived from bone marrow, the increase in circulating IgA often reflects the total body IgA burden, which is substantially contributed by secretory IgA (sIgA) produced by plasma cells in intestinal mucosa-associated lymphoid tissue. Secretory IgA serves as the first line of immune defense at mucosal surfaces by neutralizing pathogens through immune exclusion, preventing bacterial adhesion, and modulating the gut microbiota composition. Although the present study measured serum IgA rather than intestinal sIgA directly, the positive correlation between circulatory IgA levels and mucosal immune function has been well established(Gleeson et al., 2024). Future studies incorporating direct measurement of intestinal secretory IgA would further clarify the role of GLE in enhancing mucosal immunity. The absence of changes in spleen and thymus weights and the lack of significant IgM and IgY responses suggest that GLE exerts selective effects on specific immune compartments, primarily influencing mucosal rather than systemic immunity. This integrated perspective demonstrates that GLE's dual modulation of innate (antioxidant enzyme upregulation, selective cytokine reduce) and adaptive (bursal development, IgA production) immune components contributes to the improved overall immune status and growth performance observed in the GLE125 group.

For microbiome analysis, the shared core microbiota across all dietary groups indicates that GLE supplementation, regardless of the inclusion level, maintained the overall microbial stability in the cecum. The unchanged α-diversity indices suggest that GLE did not disrupt the fundamental microbial richness or evenness. This preservation of microbial ecological balance is a critical indicator of gut health, aligning with ecological theory that microbiome stability arises from balanced interactions that limit drastic shifts while allowing for adaptive responses to dietary substrates (Coyte et al., 2015). However, the clear clustering observed in the PCoA plot indicates that GLE supplementation selectively promoted specific microbial communities without altering the overall diversity. This pattern suggests that dietary garlic leaf extracts reshaped the microbiota composition rather than disrupting community stability. Notably, Firmicutes and Bacteroidetes remained the predominant phyla across all groups, which is consistent with the microbiota profiles commonly reported in healthy poultry (Aruwa et al., 2021).

Interestingly, STAMP analysis revealed that the significant alteration of *Lachnoclostridium* across all GLE-supplemented groups is particularly noteworthy. Notably, a high abundance of *Lachnoclostridium* has been linked to favorable metabolic profiles and increased market weights in poultry, suggesting that GLE may enhance the metabolic efficiency of the cecal ecosystem (Lei et al., 2022; Yang et al., 2023). This is likely driven by the organosulfur compounds in garlic, particularly allicin, which exerts potent antimicrobial and anti-inflammatory effects. Allicin has been shown to modulate the gut environment by inhibiting the nuclear factor-kappa B (NF-κB) signaling pathway, thereby reducing the production of pro-inflammatory cytokines such as IL-6 and TNF-α(Abd El-Ghany, 2024; Abd-ELrahman, et al., 2022). Such an anti-inflammatory milieu may selectively favor the proliferation of beneficial taxa while suppressing certain core anaerobic bacteria like Bacteroides. The dose-dependent enrichment of specific functional genera further underscores the targeted impact of GLE. The dose-dependent response observed in the STAMP analysis provides further insight into the selective modulation of the gut microbiota. In the low-dose group (GLE125), the enrichment of *Muribaculaceae* and *Romboutsia* indicates an adaptive shift in carbohydrate degradation and energy harvesting pathways (Yan et al., 2025). Interestingly, the significant influence on *Akkermansia* in the GLE250 group is a critical finding. *Akkermansia* is well-recognized for its role in maintaining gut microbiota homeostasis, modulating host immune functions, and strengthening the intestinal mucosal barrier (Mo et al., 2024; Tingler and Engevik, 2025). This suggests that an intermediate dose of GLE may specifically support intestinal integrity. Furthermore, the significant enrichment of Parabacteroides and members of the *Rikenellaceae* genera in the GLE500 group reflects a more robust restructuring of the microbial community toward specialized fermentation. *Parabacteroides* and *Rikenellaceae* genera are key players in the production of short-chain fatty acids (SCFAs)(Cui et al., 2022), which are essential for providing energy to colonocytes and maintaining a stable, health-associated cecal ecosystem. These observations align with ecological theory indicating that microbiome stability arises from balanced microbial interactions, where dietary substrates selectively promote specific functional communities without disrupting overall community stability. Collectively, these findings reveal that GLE selectively modulated microbial composition, favoring taxa associated with enhanced metabolic health and immune modulation in broiler chickens.

In the liver, GLE significantly upregulated *SOD* and *CAT* expression, with *SOD* increased in a dose-dependent manner and *CAT* reaching a plateau at low doses, while *GPX* expression was unchanged. This indicates that GLE activates hepatic antioxidant defense mainly through the *SOD*/*CAT* pathway, which is consistent with the regulatory effect of plant active ingredients on the Nrf2 signaling pathway (Li et al., 2024). For inflammatory genes, GLE selectively inhibited *IL-1β* and *TNF-α* expression without affecting *IL-10* and *IL-12*, showing a targeted anti-inflammatory effect. The "decrease followed by partial recovery" pattern of pro-inflammatory cytokines may be related to the feedback regulation of the NF-κB pathway induced by garlic active components (Khan et al., 2024). In the jejunum, GLE showed similar antioxidant regulation and anti-inflammatory effect to the liver. Notably, GLE significantly improved jejunal barrier function by upregulating *OCLN* and *MUC2* expression, while *CLDN1* was downregulated and *TJAP1* remained stable. In addition, GLE upregulated *GLUT2* expression, suggesting its potential to promote intestinal nutrient absorption. These results are consistent with previous findings that plant extracts enhance intestinal integrity and nutrient utilisation by reducing oxidative stress and inflammation (Abd El-Ghany, 2024; Latek et al., 2022). Comparison between tissues revealed that GLE has a more significant dose-dependent effect on hepatic *SOD*, while jejunum showed additional regulatory effects on barrier and transport genes, which may be related to the different physiological functions and microenvironments of the two organs.

## Conclusion

The significant improvements observed in broiler growth performance can be directly linked to the synergistic effects of the plant's bioactive carbohydrates (sugars), phenolics, flavonoids, and their resulting antioxidant and immunomodulatory capacities. Garlic leaf extract (GLE), particularly at 125 mg/kg, effectively improved growth performance in broiler chickens while maintaining normal physiological and blood biochemical parameters. The supplementation enhanced intestinal morphology, immune response, antioxidant capacity, and beneficially modulated the gut microbial structure, indicating improved overall health and nutrient utilisation. These effects are likely attributed to the bioactive compounds in garlic leaves with antimicrobial, antioxidant, and immunomodulatory properties. Collectively, the findings support GLE as a safe and effective phytogenic feed additive, highlighting its potential to enhance growth performance and intestinal health in broiler production. Further studies incorporating direct comparisons with AGPs are needed to evaluate its relative efficacy.

## Credit author statement

Conceptualization, C.L., S.R.S., and R.M.; methodology, C.L., Y.W., S.R.S., C.H. K., R.M., and P.S.; investigation, Y.W., C.L., P.S. A.C.M., A.M.D., and R.M.; data curation and validation, Y.W., C.L., C.H. K., S.R.S and R.M.; formal analysis, C.L., Y.W., S.R.S., A.C.M., A.M.D., and P.S.; software and visualization and C.L., Y.W., S.R.S., and P.S.; resources, C.L., S.R.S. and R.M.; project administration and funding acquisition, C.L., and S.R.S., supervision, C.L., and S.R.S.; writing—original draft preparation, Y.W., C.L., P.S., and S.R.S., writing—review and editing, C.L., C.H. K., and S.R.S. All authors have read and agreed to the published version of the manuscript.

## Funding

This research was partially supported by the Feature Areas Research Center Program within the framework of the Higher Education Sprout Project by the Ministry of Education (MOE-115-S-0023-A) in Taiwan. This research project was also supported by Fundamental Fund 2025, Chiang Mai University, Thailand.

## Disclosures

The authors declare that they have no known competing financial interests or personal relationships that could have appeared to influence the work reported in this paper.

## References

[bib0001] Abd El-Ghany W.A. (2024). Potential effects of garlic (Allium sativum L.) on the performance, immunity, gut health, anti-oxidant status, blood parameters, and intestinal microbiota of poultry: an updated comprehensive review. Animals.

[bib0002] Abd-ELrahman S.M., Mohamed S.A.-A., Mohamed S.E., El-Khadragy M.F., Dyab A.K., Hamad N., Safwat M.M., Nasr A.A., Alkhaldi A.A., Gareh A. (2022). Comparative effect of allicin and alcoholic garlic extract on the morphology and infectivity of Eimeria tenella oocysts in chickens. Animals.

[bib0003] Amad A.A., Männer K., Wendler K.R., Neumann K., Zentek J. (2011). Effects of a phytogenic feed additive on growth performance and ileal nutrient digestibility in broiler chickens. Poult. Sci..

[bib0004] Ari M., Barde R., Ogah D., Agade Y., Yusuf N., Hassan I., Muhammed M. (2012). Utilisation Of Garlic (Allium Sativum L) as a supplementary phytogenic feed additive for broilers fed commercial feeds. Egypt. Poult. Sci..

[bib0005] Aruwa C.E., Pillay C., Nyaga M.M., Sabiu S. (2021). Poultry gut health - microbiome functions, environmental impacts, microbiome engineering and advancements in characterization technologies. J. Anim. Sci. Biotechnol..

[bib0006] Awad W.A., Ghareeb K., Abdel-Raheem S., Böhm J. (2009). Effects of dietary inclusion of probiotic and synbiotic on growth performance, organ weights, and intestinal histomorphology of broiler chickens. Poult. Sci..

[bib0007] Callahan B.J., Sankaran K., Fukuyama J.A., McMurdie P.J., Holmes S.P. (2016). Bioconductor workflow for microbiome data analysis: from raw reads to community analyses. F1000Res.

[bib0008] Chen J., Wang F., Yin Y., Ma X. (2021). The nutritional applications of garlic (Allium sativum) as natural feed additives in animals. PeerJ.

[bib0009] Coyte K.Z., Schluter J., Foster K.R. (2015). The ecology of the microbiome: networks, competition, and stability. Science.

[bib0010] Cui Y., Zhang L., Wang X., Yi Y., Shan Y., Liu B., Zhou Y., Lü X. (2022). Roles of intestinal Parabacteroides in human health and diseases. FEMS Microbiol. Lett..

[bib0011] Daneshmand A., Kermanshahi H., Danesh Mesgaran M., King A.J., Ibrahim S.A. (2017). Effect of purine nucleosides on growth performance, gut morphology, digestive enzymes, serum profile and immune response in broiler chickens. Br. Poult. Sci..

[bib0012] Elagib H.A.A., El-Amin W.I.A., Elamin K.M., Malik H.E.E. (2013). Effect of dietary garlic (Allium sativum) supplementation on broiler performance and blood profile. J. Anim. Sci. Adv..

[bib0013] ELTAZI S., KA M., MA M. (2014). Effect of using garlic powder as natural feed additive on performance and carcass quality of broiler chicks. Assiut Vet. Med. J..

[bib0014] García V., Catalá-Gregori P., Hernández F., Megías M.D., Madrid J. (2007). Effect of formic acid and plant extracts on growth, nutrient digestibility, intestine mucosa morphology, and meat yield of broilers. J. Appl. Poult. Res..

[bib0015] Gleeson P.J., Camara N.O.S., Launay P., Lehuen A., Monteiro R.C. (2024). Immunoglobulin a antibodies: from protection to harmful roles. Immunol. Rev..

[bib0016] Habte-Tsion H.-M., Ge X., Liu B., Xie J., Ren M., Zhou Q., Miao L., Pan L., Chen R. (2015). A deficiency or an excess of dietary threonine level affects weight gain, enzyme activity, immune response and immune-related gene expression in juvenile blunt snout bream (Megalobrama amblycephala). Fish Shellfish Immunol..

[bib0017] Khamsaw P., Sangta J., Chaiwan P., Rachtanapun P., Sirilun S., Sringarm K., Thanakkasaranee S., Sommano S.R. (2022). Bio-circular perspective of citrus fruit loss caused by pathogens: occurrences, active ingredient recovery and applications. Horticulturae.

[bib0018] Khamsaw P., Sommano S.R., Wongkaew M., Willats W.G.T., Bakshani C.R., Sirilun S., Sunanta P. (2024). Banana Peel (Musa ABB cv. Nam Wa Mali-Ong) Source Value-Adding Compon. Funct. Prop. Bioact. Ingred..

[bib0019] Khan M.Z., Li L., Wang T., Liu X., Chen W., Ma Q., Zahoor M., Wang C. (2024). Bioactive compounds and probiotics mitigate mastitis by targeting NF-κB signaling pathway. Biomolecules.

[bib0020] Khan R.U., Nikousefat Z., Tufarelli V., Naz S., Javdani M., Laudadio V. (2012). Garlic (Allium sativum) supplementation in poultry diets: effect on production and physiology. World's. Poult. Sci. J..

[bib0021] Klasing K.C. (2007). Nutrition and the immune system. Br. Poult. Sci..

[bib0022] Kothari D., Lee W.-D., Niu K.-M., Kim S.-K. (2019). The Genus Allium as Poultry Feed Additive: A Review. Animals.

[bib0023] Larionov A., Krause A., Miller W. (2005). A standard curve based method for relative real time PCR data processing. BMC Bioinform..

[bib0024] Latek U., Chłopecka M., Karlik W., Mendel M. (2022). Phytogenic compounds for enhancing intestinal barrier function in poultry–a review. Planta Med..

[bib0025] Lei J., Dong Y., Hou Q., He Y., Lai Y., Liao C., Kawamura Y., Li J., Zhang B. (2022). Intestinal microbiota regulate certain meat quality parameters in chicken. Front. Nutr..

[bib0026] Li Y., Wang K., Li C. (2024). Oxidative stress in poultry and the therapeutic role of herbal medicine in intestinal health. Antioxidants.

[bib0027] Mo C., Lou X., Xue J., Shi Z., Zhao Y., Wang F., Chen G. (2024). The influence of Akkermansia muciniphila on intestinal barrier function. Gut Pathog..

[bib0028] Mufungwe J., Moono M.B., Chimbaka I.M., Kanyinji F., Odubote I.K., Harrison S.J., Nkhuwa J. (2024). Evaluating the effects of garlic (Allium sativum) as a feed additive on the growth performance and immune response in broiler chickens. J. Agric. Biomed. Sci..

[bib45] Rehman Z.U., Munir M.T., Rehman S.U., Khan R.U., Tahir M., Qureshi M.S. (2018). Effect of garlic on the growth performance, nutrient digestibility, and gut health of broilers: A review. J. Anim. Physiol. Anim. Nutr..

[bib0029] Reisinger N., Steiner T., Nitsch S., Schatzmayr G., Applegate T.J. (2011). Effects of a blend of essential oils on broiler performance and intestinal morphology during coccidial vaccine exposure. J. Appl. Poult. Res..

[bib0030] Sangta J., Wongkaew M., Tangpao T., Withee P., Haituk S., Arjin C., Sringarm K., Hongsibsong S., Sutan K., Pusadee T., Sommano S.R., Cheewangkoon R. (2021). Recovery of polyphenolic fraction from Arabica coffee pulp and its antifungal applications. Plants.

[bib0031] Sharifi S.D., Dibamehr A., Lotfollahian H., Baurhoo B. (2012). Effects of flavomycin and probiotic supplementation to diets containing different sources of fat on growth performance, intestinal morphology, apparent metabolizable energy, and fat digestibility in broiler chickens. Poult. Sci..

[bib0032] Shini S., Zhang D., Aland R.C., Li X., Dart P.J., Callaghan M.J., Speight R.E., Bryden W.L. (2020). Probiotic Bacillus amyloliquefaciens H57 ameliorates subclinical necrotic enteritis in broiler chicks by maintaining intestinal mucosal integrity and improving feed efficiency. Poult. Sci..

[bib0033] Somamno S.R., Saratan N., Suksathan R., Pusadee T. (2016). Chemical composition and comparison of genetic variation of commonly available Thai garlic used as food supplement. J. Appl. Bot. Food Qual..

[bib0034] Sunanta P., Chung H.-H., Kunasakdakul K., Ruksiriwanich W., Jantrawut P., Hongsibsong S., Sommano S.R. (2020). Genomic relationship and physiochemical properties among raw materials used for Thai black garlic processing. Food Sci. Nutr..

[bib0035] Sunanta P., Kontogiorgos V., Leksawasdi N., Phimolsiripol Y., Wangtueai S., Wongkaew M., Sommano S.R. (2023). Loss assessment during postharvest and handling of Thai garlic used for processing. Horticulturae.

[bib0036] Sunanta P., Kontogiorgos V., Pankasemsuk T., Jantanasakulwong K., Rachtanapun P., Seesuriyachan P., Sommano S.R. (2023). The nutritional value, bioactive availability and functional properties of garlic and its related products during processing. Front. Nutr..

[bib0037] Sunanta P., Sommano S.Rose, Luiten C.A., Ghofrani M., Sims I.M., Bell T.J., Carnachan S.M., Hinkley S.F.R., Kontogiorgos V. (2024). Fractionation and characterisation of pectin-rich extracts from garlic biomass. Food Chem..

[bib0038] Sunanta P., Sombat T., Moaphadungkul J., Chaemthet S., Nagle M., Bakshani C., Willats W., Sangta J., Sommano S. (2024). Evaluation of value adding components from postharvest biomass of Thai medicinal cannabis var. Hang Kra Rog Phu Phan. J. Appl. Res. Med. Aromat. Plants.

[bib0039] Tingler A.M., Engevik M.A. (2025). Breaking down barriers: is intestinal mucus degradation by Akkermansia muciniphila beneficial or harmful?. Infect. Immun..

[bib0040] Toghyani M., Toghyani M., Gheisari A., Ghalamkari G., Eghbalsaied S. (2011). Evaluation of cinnamon and garlic as antibiotic growth promoter substitutions on performance, immune responses, serum biochemical and haematological parameters in broiler chicks. Livest. Sci..

[bib0041] Wang J., Deng L., Chen M., Che Y., Li L., Zhu L., Chen G., Feng T. (2024). Phytogenic feed additives as natural antibiotic alternatives in animal health and production: A review of the literature of the last decade. Anim. Nutr..

[bib0042] Windisch W., Schedle K., Plitzner C., Kroismayr A. (2008). Use of phytogenic products as feed additives for swine and poultry. J. Anim. Sci..

[bib0043] Yan K., Sun X., Wang X., Zheng J., Yu H. (2025). Gut microbiota and metabolites: biomarkers and therapeutic targets for diabetes mellitus and its complications. Nutrients.

[bib0044] Yang S., Yang Y., Long X., Li H., Zhang F., Wang Z. (2023). Integrated analysis of the effects of Cecal microbiota and serum metabolome on market weights of Chinese Native Chickens. Anim. (Basel).

